# Silicon improves the photosynthetic performance of oat leaves infected with *Puccinia graminis* f. sp. *avenae*

**DOI:** 10.3389/fpls.2022.1037136

**Published:** 2022-11-23

**Authors:** Yinghao Li, Jinghui Liu, Pin Lv, Junzhen Mi, Baoping Zhao

**Affiliations:** Science Innovation Team of Oats, Inner Mongolia Agricultural University, Hothot, China

**Keywords:** oat, stem rust, silicon, photosynthesis, photosystem II

## Abstract

Stem rust, caused by *Puccinia graminis* f. sp. *avenae* (*Pga*) is a key disease affecting oat production worldwide. Silicon (Si) plays an essential role in enhancing plant resistance against pathogens. However, the scientific evidence of Si-mediated stem rust resistance of oat from the photosynthetic perspective has not been reported. The specific objective of this research was to investigate the effects of Si application on disease inhibition, photosynthetic gas exchange parameters, light response parameters, photosynthetic pigments and chlorophyll fluorescence parameters under *Pga* infection. Our results illustrated that Si application significantly reduced rust severity while the other parameters like net photosynthetic rate (*P*_n_), stomatal conductance (*Gs*), intercellular CO_2_ concentration (*C*_i_) and transpiration rate (*T*_r_) were significantly increased. Si application increased maximum photosynthetic rate (*P*_nmax_) and light saturation point (LSP), while reduced the dark respiration rate (Rd) and light compensation point (LCP). The results also indicated that Si application significantly increased the activities of maximum fluorescence (*F*_m_), variable fluorescence (*F*_v_), maximum quantum yield of photosystem II (*F*_v_/*F*_m_), photochemical quenching (qP), photosynthetic performance index (*PI*_ABS_), actual PSII quantum yield (ΦPSII), electron transfer rate (ETR), the absorbed light energy per unit reaction center (ABS/RC) and the dissipated energy per unit reaction center (DIo/RC), whereas it decreased the minimal fluorescence (*F*_o_), non-photochemical quenching (NPQ), the absorbed light energy used for electron transfer per unit reaction center (ETo/RC) and the absorbed light energy used for reduction of QA per unit reaction center (TRo/RC). The contents of chlorophyll a, b and carotenoids were also increased due to the change in the activity of parameters due to Si application as mentioned above. In conclusion, the results of the current study suggests that Si imparts tolerance to the stem rust possibly by the underlying mechanisms of improving gas exchange performance, and efficiency of the photochemical compounds in oat leaves.

## Introduction

Oat (*Avena sativa* L.) is a vital grain and forage crop, and is the sixth most important crop grown globally (Zhao et al., 2020). Oat is increasingly being used for human consumption as a beneficial health food, as it contains a variety of nutrient-rich substances, including β-glucans and vitamin E (Alminger and Eklund-Jonsson, 2008; Nazare et al., 2009; Whitehead et al., 2014). Like other agricultural crops, its production is also affected by various factors including biotic and abiotic stresses. It has been estimated that, about 25% of annual crop losses are caused by biotic stresses especially plant diseases (Lugtenberg, 2015).

Among various plant diseases, stem rust of oats is one of the most significant factors, limiting the high yield potential of the cultivars, *Puccinia graminis* f. sp. *avenae* (*Pga*) is the causative agent of oat stem rust, a major oat disease that can lead to total crop failure during severe epidemics. It is an economically important disease in the USA and Canada’s prairie provinces (Gold Steinberg et al., 2005). After infected with stem rust, the thousand-grain weight is reduced, the flour is black, and the stems are easy to break (Yuan et al., 2014). Therefore, effective prevention and control methods of stem rust are urgently needed.

Silicon (Si) has gained more attention due to its role in plant growth and imparting tolerance to various biotic and abiotic stresses (Epstein, 2009; Frew et al., 2018). Its content in the earth’s crust is about 28%, which makes it the second largest element in terms of abundance after oxygen. Due to its potential role in improving plants’ defenses against various diseases caused by fungi, bacteria and viruses, it has become a prime focus of research, especially with the increase in the pest and diseases incidence due to climate change (Debona et al., 2017; Luyckx et al., 2017).

The leaves of host plants are the major photosynthetic tissues and are the main targets of many pathogens. The pathogens infection directly reduces the photosynthetic performance of the leaves and eventually results in huge losses in terms of crop yield (Yang et al., 2014). Inhibition of photosynthesis by pathogens has been reported in many plants. Studies have shown that the photosynthetic activities of wheat are closely related to stripe rust, and the photosystem II (PSII) is highly susceptible to *Puccinia striiformis* f. sp. *tritici* (*Pst*) infection, they also found that wheat may effectively improve resistance to stripe rust by maintaining a higher PSII activity. This result provides a better understanding of wheat resistance mechanisms against stripe rust infections (Chen et al., 2015; Li et al., 2015). Some evidence suggests that, within given limits, Si may maintain the photosynthetic rate of plants upon pathogen infection (Aucique-Pe´rez et al., 2014; Domiciano et al., 2015; Tatagiba et al., 2015; Debona et al., 2017), but the underlying mechanisms remain unresolved. Hence, the current studies were aimed to explore Si’s protective role and underlying defense response mechanism in oat against *Pga* infection.

We hypothesized that Si-modulated operational ability of photosystem II may be a potential mechanism imparting stem rust resistance in oat. The objective of the current study was to analyze the photosynthetic characteristics and photosystem II functions in leaves to reveal the photosynthetic mechanism of Si induced resistance towards stem rust in oat, to test the original hypothesis.

## Materials and methods

### Materials, culture conditions and experimental design

Oat cultivar Bayou 1 (high susceptible) was used for inoculation with *Pga* (race TKR, was provided by the Institute of Plant Immunity, Shenyang Agricultural University). Twenty oat seeds were grown in a 12 cm diameter pot (12cm×15cm) with the peat soil matrix, seedlings were cultured in a greenhouse (20 ± 2°C with a photoperiod of 16 h light/8 h dark) at the Oat Research Center of Inner Mongolia Agricultural University.

Four treatments with three replications each were prepared for both plants: (1) CK (no silicon and no *Pga* inoculation); (2) +Si-P (1.5 mmol·L^–1^ silicon application, no *Pga* inoculation); (3) -Si+P (no silicon, *Pga* inoculation); (4) +Si+P (1.5 mmol·L^–1^ silicon application and *Pga* inoculation). Silicon (1.5 mmol·L^–1^) was added as potassium silicate (K_2_SiO_3_) solution, in the silicon-deficient treatment, potassium chloride (KCl, pH 5.5) was used to equal the potassium component of the Si treatment, and the nutrient solution used was configured according to Hoagland’s classic formula (Jiang et al., 2013). From the beginning of the emergence of oat seedlings, different treatments of nutrient solutions were used to irrigate the pots every 3 days, 150 mL per pot. Below the pot, a solution collector plate was added and the cultivation system was opened.

The inoculation was carried out when the oat seedlings grew to the two-leaf stage (one leaf and one sprout). The method of inoculation was carried out as described by Li et al. (2014). First, the leaves were sprayed with a 0.05% Tween-20 solution (Polyoxyethylene sorbaitan monolaurate, water soluble emulsifier, 0.05%) using a handheld atomizer to form a water film on the leaves. Then, flat toothpick (only by contact) was used to pick fresh urediniospores (0.01 g) and inoculated on the seedlings. Finally, the inoculated plants were kept in a mist chamber at 18 to 20°C for 16 h in darkness. Plants were transferred to a 16/8 h (light/dark) photoperiod, and a climatic chamber at 24 °C with 80 ± 5% humidity.

### Photosynthetic gas exchange parameters

The leaf gas exchange parameters of all treatment combination were recorded at 0, 1, 3, 5, 7, 9 and 11days after inoculation. The readings of the variables were performed on the first fully expanded leaf (the intermediate section, the chlorotic area), three readings were taken per leaf in the pot.

The net photosynthetic rate (*P*_n_), stomatal conductance (*G*_s_), intercellular CO_2_ concentration (*C*_i_) and transpiration rate (*T*_r_) were measured at room temperature (25°C) and 60% relative humidity with a portable system (CIRAS-3, PP Systems, UK). The photosynthetic active radiation (PAR) in the leaf chamber, provided by the CIRAS-3 LED light source, was set to 1000 µmol·m^–2^s^–1^.

On day 11 after inoculation, under a fixed atmospheric CO_2_ concentration (*C*_a_) of 380 μmol·mol^-1^, the net photosynthetic rate (*P*_n_), stomatal conductance (*G*_s_) and intercellular CO_2_ concentration (*C*_i_) to photosynthetic active radiation (PAR) curves of the most fully expanded leaf was recorded after the PAR of LED light sourced couple to a leaf chamber were set to 2000, 1800, 1500, 1200, 1000, 800, 500, 200, 100, 50, 20 and 0 μmol·m^−2^s^−1^, respectively. The maximum photosynthetic rate (*P*_nmax_) and dark respiration rate (Rd) were recorded. The light compensation point (LCP) was calculated when PAR was close to zero and the light saturation point (LSP) as the PAR value was obtained when photosynthesis reached *P*_nmax_.

### Chlorophyll a fluorescence parameters

After the photosynthetic gas exchange parameter measurements, a chlorophyll fluorescence experiment was carried out on the same leaves using a plant efficiency analyzer (Handy-PEA, Hansatech, UK). Leaves were dark-adapted for 30 min before measurements, during light illumination, chlorophyll a fluorescence intensity in dark- adapted leaves rose rapidly from an initial minimal level, *F*_o_ (O step) to the maximal level, *F*_m_ (P step), and two intermediate steps designated as J and I appeared at 2 and 30 ms, respectively. So, a fast rise of the chlorophyll fluorescence, transient with the notation OJIP, was recorded. The photosynthetic performance index based on the absorbed light energy (*PI*_ABS_), the absorbed light energy per unit reaction center (ABS/RC), the absorbed light energy used for reduction of QA per unit reaction center (TRo/RC), the absorbed light energy used for electron transfer per unit reaction center (ETo/RC), and the dissipated energy per unit reaction center (DIo/RC) were obtained. The maximum quantum yield of PSII (*F*_v_/*F*_m_) was calculated according to the formula: *F*_v_/*F*_m_= [(*F*_m_-*F*_o_)/*F*_m_].

Modulated chlorophyll fluorescence was measured with a FMS-2 pulse-modulated fluorometer (Hansatech, UK). The light-adapted leaves were continuously illuminated by actinic light at 800 μmol·m^−2^s^−1^ from the FMS-2 light source (PFD), steady-state fluorescence (*F*_s_) was recorded after a 2 min illumination, and a saturation pulse (8000 μmol·m^-2^s^-1^; 0.8s) was applied to achieve the light-adapted maximum fluorescence (*F*_m_’). The actinic light was then turned off, and the minimum fluorescence in the light-adapted state (*F*_o_’) was determined by a 3 s illumination with far-red light. The following parameters were calculated (Maxwell and Johnson, 2000):

Actual PSII quantum yield, ΦPSII = (*F*_m_’-*F*_s_)/*F*_m_’
Electron transport rate, ETR = ΦPSII×PFD×0.5×0.84
Photochemical quenching, qP= (*F*_m_’- *F*_s_)/(*F*_m_’-*F*_o_’)
Non-photochemical quenching, NPQ = (*F*_m_/*F*_m_’)-1

### Photosynthetic pigment

The leaves of each plant per replication of each treatment were collected at 0, 1, 3, 5, 7, 9 and 11days after inoculation, 0.5 g of leaf tissue was frozen in liquid nitrogen, homogenized in 80% acetone with a small amount of SiO_2_ and centrifuged (3600 × g, 5 min). Contents of chlorophyll a (Chla), chlorophyll b (Chlb) and total carotenoids (Car) in the supernatant were then determined spectrophotometrically (UV2300IISpectrophotometer, CHINA), according to Lichtenthaler (Lichtenthaler, 1987).

### Data analysis

In this study, the charts were made using Microsoft Excel 2016 software. All data were expressed as the mean ± SE. One-way ANOVA was performed to test the significance of the observed differences using SPSS (Inc., Chicago, USA). Differences between parameters were evaluated using Duncan’ s method, and *P ≤* 0.05 was considered the statistically significant threshold.

## Results

### Phenotypes

**Figure 1:**
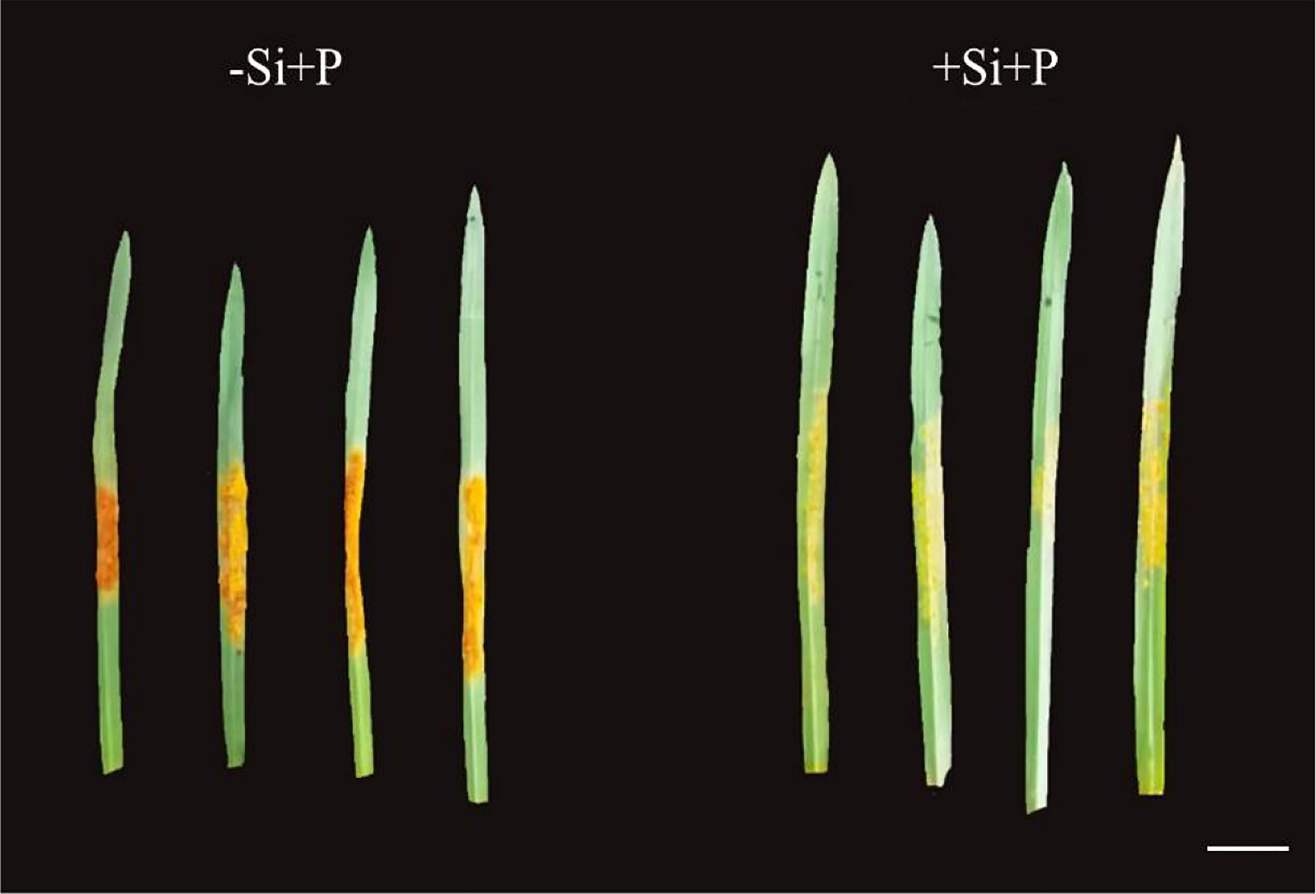
Effects of Si application and *Pga* inoculation on phenotype of oat, scale bar=1 cm. -Si+P, no Si application and *Pga* inoculation; +Si+P, 1.5 mmol·L^–1^ Si application and *Pga* inoculation.

Obviously, 15 days after inoculation with *Pga*, leaves developed many orange pustules without Si, and the color depth of pustules was significantly reduced under Si application (**Figure 1**).

### Photosynthetic gas exchange parameters

**Figure 2:**
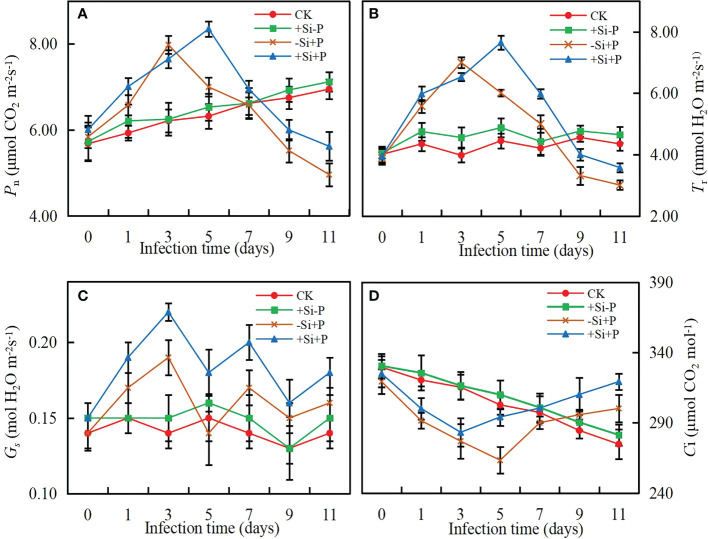
Effects of Si application and *Pga* inoculation on photosynthetic parameters of oat leaves. **(A)**, net photosynthetic rate (*P*_n_); **(B)**, stomatal conductance (*G*_s_); **(C)**, transpiration rate (*T*_r_); **(D)**, intercellular CO_2_ concentration (*C*_i_). CK, no Si application and no *Pga* inoculation; +Si-P, 1.5 mmol·L^–1^ Si application and no *Pga* inoculation; -Si+P, no Si application and *Pga* inoculation; +Si+P, 1.5 mmol·L^–1^ Si application and *Pga* inoculation.

The values of photosynthetic parameters showed no significant difference for non-inoculated oat seedlings (**Figure 2**). In the early stage of infection (0-3 days after inoculation), *P*_n_ (**Figure 2A**) and *T*_r_ (**Figure 2B**) increased rapidly, then began to decline gradually after reaching the peak and *C*_i_ (**Figure 2D**) exhibited the opposite tendency, while *G*_s_ (**Figure 2C**) showed a chaotic change pattern. Compared with -Si+P, Si application (+Si+P) increased *P*_n_, *T*_r_, *G*_s_ and *C*_i_ significantly (*P ≤* 0.05).

**Figure 3:**
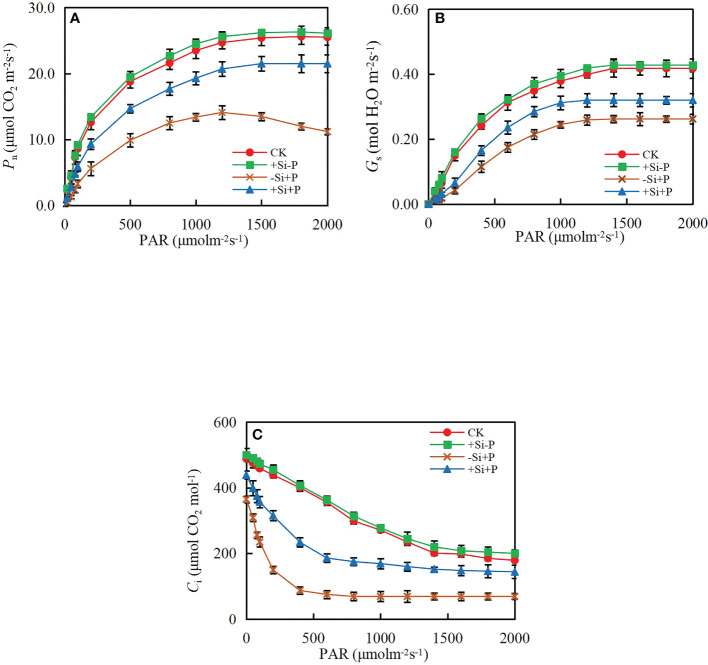
Response of **(A)** net photosynthetic rate (*P*_n_), **(B)** stomatal conductance (*G*_s_) and **(C)** intercellular CO_2_ concentration (*C*_i_) to photosynthetic photon flux density (PAR) of oat leaves. CK, no Si application and no *Pga* inoculation; +Si-P, 1.5 mmol·L^–1^ Si application and no *Pga* inoculation; -Si+P, no Si application and *Pga* inoculation; +Si+P, 1.5 mmol·L^–1^ Si application and *Pga* inoculation.

Without *Pga* inoculation, *P*_n_ (**Figure 3A**) and *G*_s_ (**Figure 3B**) gradually increased with PAR, while *C*_i_ (**Figure 3C**) gradually decreased, and finally remained stable. Under -Si+P treatment, *P*_n_ began to decrease gradually when it reached the highest point, while remained stable under +Si+P treatment.

**Figure 4:**
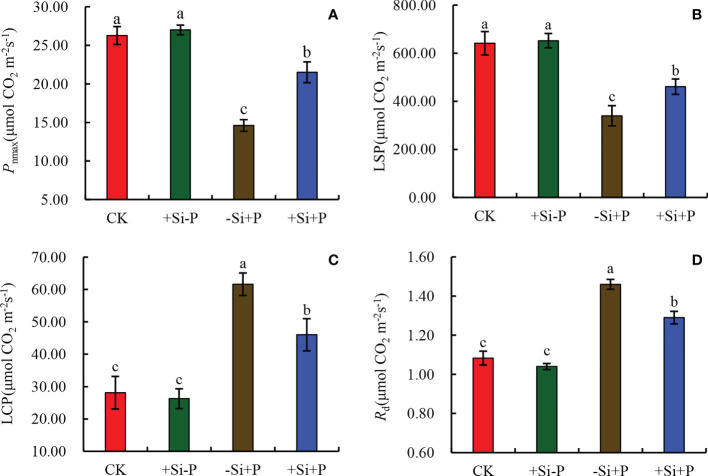
Effects of Si application and *Pga* inoculation on light response parameters of oat leaves. **(A)**, maximum photosynthetic rate (*P*_nmax_); **(B)**, light saturation point (LSP); **(C)**, light compensation point (LCP); **(D)**, dark respiration rate (Rd). CK, no Si application and no *Pga* inoculation; +Si-P, 1.5 mmol·L^–1^ Si application and no *Pga* inoculation; -Si+P, no Si application and *Pga* inoculation; +Si+P, 1.5 mmol·L^–1^ Si application and *Pga* inoculation. Data are expressed as mean ± SE (n=3). According to Duncan’s multiple comparison tests among treatments, different letters on bars show significant differences at 0.05 level of probability.

By fitting the *P*_n_/PAR curve of oat leaves, various light response parameters under different treatments were obtained (**Figure 4**). In non-inoculated oat seedlings, Si had no effect on light response parameters. Compared with CK, *P*_nmax_ (**Figure 4A**) and LSP (**Figure 4B**) were significantly decreased by 44.42 and 47.14%, respectively, while LCP (**Figure 4C**) and Rd (**Figure 4D**) were significantly increased by 119.30 and 35.19% under -Si+P treatment, respectively; Compared with -Si+P, Si application (+Si+P) significantly increased *P*_nmax_ and LSP by 47.26 and 15.30%, respectively, while decreased LCP and Rd by 25.28 and 11.64%, respectively (*P ≤* 0.05).

### Photosynthetic pigments

**Figure 5:**
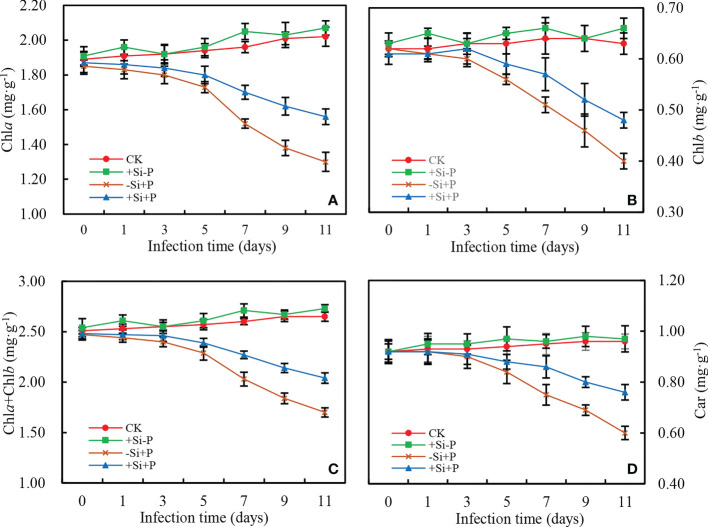
Effects of Si application and *Pga* inoculation on photosynthetic pigment content of oat leaves. **(A)**, chlorophyll a (Chla); **(B)**, chlorophyll b (Chlb); **(C)**, chlorophyll a+b (Chla+Chlb); **(D)**, total carotenoids (Car). CK, no Si application and no *Pga* inoculation; +Si-P, 1.5 mmol·L^–1^ Si application and no *Pga* inoculation; -Si+P, no Si application and *Pga* inoculation; +Si+P, 1.5 mmol·L^–1^ Si application and *Pga* inoculation.

In non-inoculated oat seedlings, Si application did not have any effects on the content of pigments (Chla, Chlb, Car and Chla+Chlb) in oat leaves (**Figure 5**). Pigments content began to decrease significantly at 3 d after *Pga* inoculation, and compared with -Si+P, Si application (+Si+P) significantly increased Chla (up to 20.00%) (**Figure 5A**), Chlb (up to 20.00%) (**Figure 5B**), Car (up to 26.67%) (**Figure 5D**) and Chla+Chlb (up to 20.00%) (**Figure 5C**), respectively (*P ≤* 0.05).

### Chlorophyll fluorescence parameters

**Figure 6:**
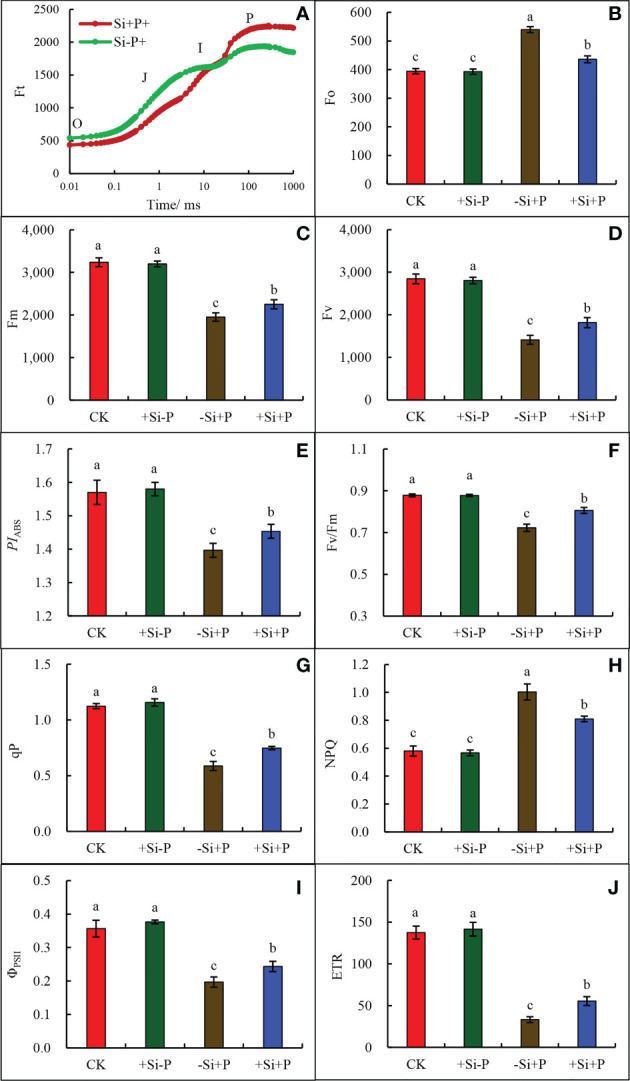
Effects of Si application and *Pga* inoculation on OJIP curves and Chlorophyll fluorescence parameters of oat leaves. **(A)**, OJIP curve; **(B)**, chlorophyll b (*F*_o_); **(C)**, maximum fluorescence (*F*_m_); **(D)**, variable fluorescence (*F*_v_); **(E)**, photosynthetic performance index (*PI*_ABS_); **(F)**, maximum quantum yield of PSII (*F*_v_/*F*_m_); **(G)**, Photochemical quenching (qP); **(H)**, non-photochemical quenching (NPQ); **(I)**, actual PSII quantum yield (ΦPSII); **(J)** electron transport rate (ETR). CK, no Si application and no *Pga* inoculation; +Si-P, 1.5 mmol·L^–1^ Si application and no *Pga* inoculation; -Si+P, no Si application and *Pga* inoculation; +Si+P, 1.5 mmol·L^–1^ Si application and *Pga* inoculation. Data are expressed as mean ± SE (n=3). According to Duncan’s multiple comparison tests among treatments, different letters on bars show significant differences at 0.05 level of probability.

As observed in the study, under *Pga* infection conditions, Si application (+Si+P) lead to the decrease of *F*_o_ (**Figure 6B**) and the increase of *F*_m_ (**Figure 6C**) and changed the trend of OJIP curve (**Figure 6A**).

Compared with CK, *Pga* inoculation (-Si+P) significantly reduced *F*_m_ (**Figure 6C**), *F*_v_ (**Figure 6D** ), *PI*_ABS_ (**Figure 6E**), *F*_v_/*F*_m_ (**Figure 6F**), qP (**Figure 6G**), ΦPSII (**Figure 6I**) and ETR (**Figure 6J**), which were reduced by 39.7, 50.4, 10.8,18.2, 47.3, 44.4 and 75.9% respectively, while *F*_o_ (**Figure 6B**) and NPQ (**Figure 6H**) were increased by 36.8 and 71.4%, respectively.

Compared with -Si+P, Si application (+Si+P) increased *F*_m_, *F*_v_, *F*_v_/*F*_m_, *PI*_ABS_, ETR, qP and ΦPSII by 15.4, 28.6, 12.5, 3.6, 67.0, 27.1 and 20.0%, respectively, while *F*_o_ and NPQ were significantly decreased by19.2 and 19.0% (*P ≤* 0.05).

**Figure 7:**
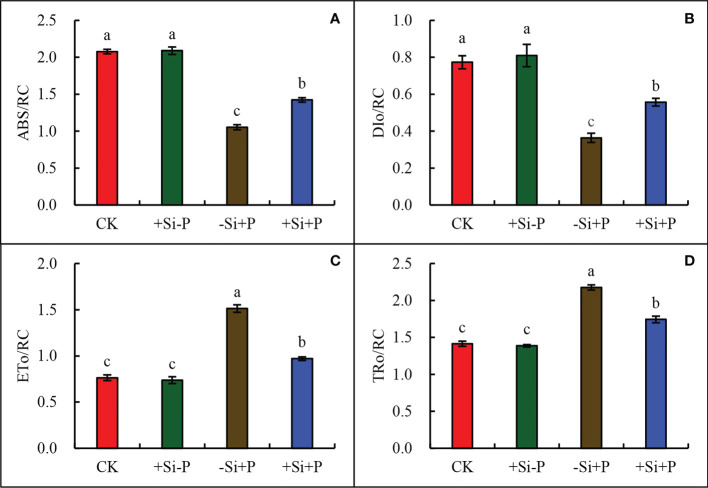
Effect of Si application and *Pga* inoculation on ABS/RC **(A)**, DIo/RC **(B)**, ETo/RC **(C)**, and TRo/RC **(D)** of oat leaves. CK, no Si application and no *Pga* inoculation; +Si-P, 1.5 mmol·L^–1^ Si application and no *Pga* inoculation; -Si+P, no Si application and *Pga* inoculation; +Si+P, 1.5 mmol·L^–1^ Si application and *Pga* inoculation. Data are expressed as mean ± SE (n=3). According to Duncan’s multiple comparison tests among treatments, different letters on bars show significant differences at 0.05 level of probability.

Compared with CK, *Pga* inoculation (-Si+P) significantly reduced ABS/RC (**Figure 7A**) and DIo/RC (**Figure 7B**) by 49.5 and 53.3%, respectively; while increased ETo/RC (**Figure 7C**) and TRo/RC (**Figure 7D**) by 98.7 and 54.6%, respectively. Compared with -Si+P, Si application (+Si+P) significantly increased ABS/RC and DIo/RC by 35.2 and 55.6%, respectively, while reduced ETo/RC and TRo/RC by 35.8 and 20.2%, respectively (*P ≤* 0.05).

## Discussion

The present study showed that the leaf photosynthetic capacity sensitively responded to *Pga* infection and Si application in oat. The results demonstrated that leaf disease symptoms were remarkably reduced in oat leaves supplied with Si (**Figure 1**). Similarly, wheat could be reduced the rice blast intensity by keeping a high foliar Si concentration (Xavier-Filha et al., 2011). This also supports previous reports (Camargo et al., 2020) and several other plants against foliar pathogens (Fauteux et al., 2005; Domiciano et al., 2010; Resende et al., 2012).

Our study provides new information from a photosynthesis perspective regarding the effect of Si on improving oat tolerance to stem rust. Previous studies have shown that there are two aspects that characterize the possible mechanisms of Si imparting plant tolerance to various diseases. One is Si polymerization and deposition in epidermal cell walls below the cuticle, forming a cuticle-Si double layer in leaves to prevent fungal invasion (Kim et al., 2002; Cai et al., 2008; Hayasaka et al., 2008; Samuels et al., 2010). Another view is that Si can induce the defense responses of plants by increasing the activity of defense-related enzymes such as peroxidase, polyphenoloxidase, phenylalanine ammonia-lyase, etc., and promoting the synthesis of antifungal compounds such as phenolics and phytoalexins (Fawe et al., 1998; Rodrigues et al., 2004; Re´mus-Borel et al., 2005). Traditionally, these positive effects of Si have been associated with alleviating biotic stresses, improving resistance to lodging, and increasing leaves erectness, which allows better light transmittance through plant canopies and enhances whole-plant photosynthesis (Tamai and Ma, 2008).

Necrotrophic pathogens produce many hydrolases that degrade plant cell walls and ultimately have a profound effect on photosynthesis (Scholes and Rolfe, 2009). In our study, due to infection with stem rust, the photosynthetic activity was remarkably impaired, as also noticed on sugarcane leaves infected with brown rust (Camargo et al., 2020). Studies have found that Si increased rice resistance against brown spots was related to the promotion of the primary metabolism of photorespiration (Vivancos et al., 2015).

For the oat-*Pga* interaction, the reduction of symptoms on the leaves of plants treated with Si improved their gas exchange performance and reduction in the dysfunction at the photochemical level (Aucique-Pérez et al., 2014). Meanwhile, the results of the present study bring new evidence that the photosynthetic machinery of *Pga* infected oat leaves can be significantly protected when supplied with Si; such protection was related to some preservation of the photosynthetic performance, it is shown as the higher ability to use the incident light {higher values for *PI*_ABS_ (**Figure 6E**), ABS/RC (**Figure 7A**), DIo/RC (**Figure 7B**) and LSP (**Figure 4B**)}, as well as the partial preservation of chlorophylls and carotenoids contents (**Figure 5**). Kretschmer et al. (2020) also found that the *F. oxysporum* generally had negative effects on chlorophyll and carotenoid content in tomato leaves, which was in agreement with our view. Lee et al. (2015) observed that carotenoid or chlorophyll biosynthesis silencing at the phytoene desaturase or Mg-chetalase H steps during wheat infection by the hemibiotrophic fungus *Zymoseptoria tritici*, resulted in a faster appearance of HR symptoms.

Sampol et al. (2003) first reported that *G*_s_ was the main limiting factor on *P*_n_ in response to grapevine leaves after infected by the grapevine fan leaf virus (GFLV). To a certain extent, adding Si could indirectly have helped preserve the photosynthetic apparatus’s functionality and the gas exchange capacity upon fungal infection by decreasing rust severity, as noted by the significantly higher values of *P*_n_ (**Figure 2A**), *T*_r_ (**Figure 2B**), *G*_s_ (**Figure 2C**), and *C*_i_ (**Figure 2D**) in the Si application plants under the *Pga* infection. Regardless of whether Si is applied or not, infected plants may also negatively affect the process of CO_2_ fixation and decrease their capacity to use solar energy for photosynthesis. This result is shown by non-stomatal limitations, as indicated by declines in *P*_n_ but not *C*_i_, despite the decreases in *G*_s_ significantly. Si application can enhance plant cell wall, and cell wall thickness is one of the main factors determining the structural components of *G*_s_ because higher thickness increases the pathway of CO_2_ from the intercellular spaces to the chloroplast membrane (Yamamoto et al., 2012).

The fluorescence signal rose from the initial fluorescence level (*F*_o_) (**Figure 6B**) to the maximum level (*F*_m_) (**Figure 6C**) with well-defined intermediate J and I step, showing a typical polyphase behavior (OJIP curve) (**Figure 6A**). These results demonstrated that all samples were photosynthetically active when supplied with Si in response to *Pga*. Measurements of chlorophyll fluorescence parameters provided important information for PSII activity and changes in photosynthetic metabolism ability of infected leaves (Schnabel et al., 1998). In the current data that we referred to, there is a very little useful information available on the relationship between Si and PSII photochemical efficiency of infected leaves. Our results showed that *Pga* infection significantly reduced chlorophyll fluorescence parameters, including *F*_m_ (**Figure 6C**), *PI*_ABS_ (**Figure 6E**), *F*_v_/*F*_m_ (**Figure 6F**), qP (**Figure 6G**), and ETR (**Figure 6J**), but Si application significantly increased these parameters in the infected plants. Bassanezi et al. (2002) found that electron transport capacity such as ETR, ETo/RC, generation of ATP and NADPH did not change apparently in the healthy areas of diseased leaves, but chlorophyll fluorescence emission decreased in visibly lesioned areas of bean rust, angular leaf spot, or anthracnose. Rahoutei et al. (2010) pointed out that in both symptomatic and asymptomatic leaves of Nicotiana benthamiana Gray plants, infected with pepper mild mottle virus (PMMoV) and Paprika mild mottle virus (PaMMoV), the ETR in PSII deceased. In addition, the results of the current study showed that *Pga* infection significantly increased ETo/RC (**Figure 6J**) while it was significantly reduced after the application of Si. Bonfig et al. (2006) reported that non-photochemical quenching (NPQ) in Arabidopsis leaves was decreased after being infected with either a virulent or an avirulent strain of *Pseudomonas syringae*. However, the NPQ in oat leaves was found to be increased significantly after the infection of *Pga* (**Figure 6H**), which demands further studies to elucidate the cause of this.

In addition, the experimental results showed that the ETR (**Figure 6J**) reduction is much lower than *P*_nmax_ (**Figure 4A**), coupled with the lower LSP (**Figure 4B**), which are expected to generate a photoinhibition event. It explains the performance of the *P*_n_/PAR (**Figure 3A**) response curve reasonably in this study, especially for the infected plants without Si application treatment. In fact, the diseased plants are prone to suffer from photoinactivation, which may lead to oxidative damage and losses of PSII functionality, ultimately leading to increased *F*_o_ (**Figure 6B**) values (Baker, 2008; Rolfe and Scholes, 2012).

## Conclusions

The results from the present study indicated that Si application in oat could promote plant growth and enhance plant tolerance to stem rust and improve its photosynthetic performance. During *Pga* infection with Si addition, adequate *G*_s_ and *P*_n_ values were maintained, which helped to protect the photosynthetic system against chronic photoinhibition. Under *Pga* inoculation, Si application increased pigment content and made it more efficient in the process of light energy dissipation such as *F*_v_/*F*_m_, *PI*_ABS_, ABC/RC and DIo/RC. Our findings also suggest that gas exchange properties and photochemical functions are involved in the Si-mediated amelioration of oats to stem rust.

## Data availability statement

The original contributions presented in the study are included in the article/supplementary material. Further inquiries can be directed to the corresponding author.

## Author contributions

JL conceived and supervised the experiments. YL performed the experiments, contributed to data analysis, and wrote the paper. BZ, JM, and PL gave valuable advice for the modifications of the paper. All authors contributed to the article and approved the submitted version.

## Funding

This study was supported by National Key R&D Program of China (2018YFE0107900) and National Modern Agricultural Industry Technology System (CARS-07).

## Conflict of interest

The authors declare that the research was conducted in the absence of any commercial or financial relationships that could be construed as a potential conflict of interest.

## Publisher’s note

All claims expressed in this article are solely those of the authors and do not necessarily represent those of their affiliated organizations, or those of the publisher, the editors and the reviewers. Any product that may be evaluated in this article, or claim that may be made by its manufacturer, is not guaranteed or endorsed by the publisher.
